# Preoperative growth dynamics of untreated glioblastoma: Description of an exponential growth type, correlating factors, and association with postoperative survival

**DOI:** 10.1093/noajnl/vdae053

**Published:** 2024-04-03

**Authors:** Daniel Feucht, Patrick Haas, Marco Skardelly, Felix Behling, David Rieger, Paula Bombach, Frank Paulsen, Elgin Hoffmann, Till-Karsten Hauser, Benjamin Bender, Mirjam Renovanz, Maximilian Niyazi, Ghazaleh Tabatabai, Marcos Tatagiba, Constantin Roder

**Affiliations:** Center for Neuro-Oncology, Comprehensive Cancer Center Tübingen-Stuttgart, University Hospital Tübingen, Tübingen, Germany; Department of Neurosurgery, University Hospital Tübingen, Eberhard Karls University Tübingen, Tübingen, Germany; Center for Neuro-Oncology, Comprehensive Cancer Center Tübingen-Stuttgart, University Hospital Tübingen, Tübingen, Germany; Department of Neurosurgery, University Hospital Tübingen, Eberhard Karls University Tübingen, Tübingen, Germany; Center for Neuro-Oncology, Comprehensive Cancer Center Tübingen-Stuttgart, University Hospital Tübingen, Tübingen, Germany; Department of Neurosurgery, Klinikum am Steinenberg, Reutlingen, Germany; Center for Neuro-Oncology, Comprehensive Cancer Center Tübingen-Stuttgart, University Hospital Tübingen, Tübingen, Germany; Department of Neurosurgery, University Hospital Tübingen, Eberhard Karls University Tübingen, Tübingen, Germany; Department of Neurology and Interdisciplinary Neuro-Oncology, Hertie Institute for Clinical Brain Research, University Hospital Tübingen, Eberhard Karls University Tübingen, Tübingen, Germany; Department of Radiation Oncology, University Hospital Tübingen, Eberhard Karls University Tübingen, Tübingen, Germany; Center for Neuro-Oncology, Comprehensive Cancer Center Tübingen-Stuttgart, University Hospital Tübingen, Tübingen, Germany; Department of Neurology and Interdisciplinary Neuro-Oncology, Hertie Institute for Clinical Brain Research, University Hospital Tübingen, Eberhard Karls University Tübingen, Tübingen, Germany; Center for Neuro-Oncology, Comprehensive Cancer Center Tübingen-Stuttgart, University Hospital Tübingen, Tübingen, Germany; Department of Neurology and Interdisciplinary Neuro-Oncology, Hertie Institute for Clinical Brain Research, University Hospital Tübingen, Eberhard Karls University Tübingen, Tübingen, Germany; Center for Neuro-Oncology, Comprehensive Cancer Center Tübingen-Stuttgart, University Hospital Tübingen, Tübingen, Germany; Center for Neuro-Oncology, Comprehensive Cancer Center Tübingen-Stuttgart, University Hospital Tübingen, Tübingen, Germany; Department of Radiation Oncology, University Hospital Tübingen, Eberhard Karls University Tübingen, Tübingen, Germany; Department of Diagnostic and Interventional Neuroradiology, Department of Radiology, University Hospital Tübingen, Eberhard Karls University Tübingen, Tübingen, Germany; Center for Neuro-Oncology, Comprehensive Cancer Center Tübingen-Stuttgart, University Hospital Tübingen, Tübingen, Germany; Department of Diagnostic and Interventional Neuroradiology, Department of Radiology, University Hospital Tübingen, Eberhard Karls University Tübingen, Tübingen, Germany; German Cancer Consortium (DKTK), DKFZ partner site Tübingen, Tübingen, Germany; Center for Neuro-Oncology, Comprehensive Cancer Center Tübingen-Stuttgart, University Hospital Tübingen, Tübingen, Germany; Department of Neurosurgery, University Hospital Tübingen, Eberhard Karls University Tübingen, Tübingen, Germany; Department of Neurology and Interdisciplinary Neuro-Oncology, Hertie Institute for Clinical Brain Research, University Hospital Tübingen, Eberhard Karls University Tübingen, Tübingen, Germany; Center for Neuro-Oncology, Comprehensive Cancer Center Tübingen-Stuttgart, University Hospital Tübingen, Tübingen, Germany; Department of Radiation Oncology, University Hospital Tübingen, Eberhard Karls University Tübingen, Tübingen, Germany; Center for Neuro-Oncology, Comprehensive Cancer Center Tübingen-Stuttgart, University Hospital Tübingen, Tübingen, Germany; Department of Neurology and Interdisciplinary Neuro-Oncology, Hertie Institute for Clinical Brain Research, University Hospital Tübingen, Eberhard Karls University Tübingen, Tübingen, Germany; German Cancer Consortium (DKTK), DKFZ partner site Tübingen, Tübingen, Germany; Center for Neuro-Oncology, Comprehensive Cancer Center Tübingen-Stuttgart, University Hospital Tübingen, Tübingen, Germany; Department of Neurosurgery, University Hospital Tübingen, Eberhard Karls University Tübingen, Tübingen, Germany; Center for Neuro-Oncology, Comprehensive Cancer Center Tübingen-Stuttgart, University Hospital Tübingen, Tübingen, Germany; Department of Neurosurgery, University Hospital Tübingen, Eberhard Karls University Tübingen, Tübingen, Germany

**Keywords:** glioblastoma, MR imaging, neurosurgery, survival, tumor growth rate

## Abstract

**Background:**

Little is known about the growth dynamics of untreated glioblastoma and its possible influence on postoperative survival. Our aim was to analyze a possible association of preoperative growth dynamics with postoperative survival.

**Methods:**

We performed a retrospective analysis of all adult patients surgically treated for newly diagnosed glioblastoma at our center between 2010 and 2020. By volumetric analysis of data of patients with availability of ≥3 preoperative sequential MRI, a growth pattern was aimed to be identified. Main inclusion criterion for further analysis was the availability of two preoperative MRI scans with a slice thickness of 1 mm, at least 7 days apart. Individual growth rates were calculated. Association with overall survival (OS) was examined by multivariable.

**Results:**

Out of 749 patients screened, 13 had ≥3 preoperative MRI, 70 had 2 MRI and met the inclusion criteria. A curve estimation regression model showed the best fit for exponential tumor growth. Median tumor volume doubling time (VDT) was 31 days, median specific growth rate (SGR) was 2.2% growth per day. SGR showed negative correlation with tumor size (rho = −0.59, *P* < .001). Growth rates were dichotomized according to the median SGR.OS was significantly longer in the group with slow growth (log-rank: *P* = .010). Slower preoperative growth was independently associated with longer overall survival in a multivariable Cox regression model for patients after tumor resection.

**Conclusions:**

Especially small lesions suggestive of glioblastoma showed exponential tumor growth with variable growth rates and a median VDT of 31 days. SGR was significantly associated with OS in patients with tumor resection in our sample.

**Key Points**

1. Newly diagnosed glioblastoma showed near-exponential growth patterns with a median volume doubling time of 31 days.
2. Preoperative tumor growth rate was associated with overall survival in patients who underwent tumor resection and might be considered as a novel prognostic factor.

**Importance of the Study**

This study analyzed preoperative growth of glioblastoma precisely through MRI datasets with 1 mm slice thickness. Based on these findings we defined groups of slow and fast-growing glioblastoma, which showed significant differences in overall survival in Kaplan–Meier Curves. In multivariable analysis, the preoperative tumor growth rate-specific growth rate was a significant predictor for survival in patients with tumor resection, but not for the whole series. With further investigation, preoperative growth dynamics might therefore represent a new prognostic and subclassification factor in the future. These findings might enable new insights into tumor biology when combined with molecular markers and likely new aspects for the planning of surgical and postoperative treatment.

Glioblastoma is the most common primary malignant brain tumor.^1^ Patients may die within 3–4 months if left untreated. Therefore, maximum safe surgical resection with postoperative radiochemotherapy is the standard of care.^2,3^ If this is not possible due to the tumor localization, a biopsy with subsequent treatment is performed.^3^

Despite multimodal treatment options, glioblastoma remains incurable with a median overall survival (OS) of 12–15 months, even in selected clinical trial populations.^4–6^

Affected patients usually become symptomatic with headache, cognitive and/or motor deficits, aphasia, or epileptic seizures.^7^ After initial imaging diagnosis by cerebral MRI, a quick transfer to a neurosurgical center for biopsy or resection is usually performed within 1 or 2 weeks.^8^ In order to not procrastinate effective treatment for patients suffering from this aggressive fast-growing tumor, studies evaluating the preoperative growth dynamics of human glioblastoma are rare. Therefore, little is known about the natural growth dynamics of untreated and de novo diagnosed glioblastoma in vivo.

Mechanisms and dynamics of tumor growth in general have been subject of research for a long time. Serial lung X-rays of metastases demonstrated exponential growth of these as early as the 1950s.^9^ However, purely exponential growth with a constant volume doubling time (VDT) has been questioned for glioblastoma in the past with reference to the known infiltrative nature of the tumor and extension beyond contrast enhancement on MRI.^10^ Other proposed growth functions were cubic or radial-linear^11,12^ and Gompertzian,^13^ which has an initial near-exponential growth which is followed by a slower growth rate that plateaus as the size of the tumor increases. These hypotheses of growth dynamics relied on mathematical models or pathophysiological assumptions. A possible prognostic relevance of preoperative tumor growth dynamics on OS as well as on tumor progression has been discussed controversially.^14–16^ However, the available studies rely on MRI sequences with variable quality and up to 5 mm slice thickness, possibly resulting in a bias by over-estimated tumor sizes caused by interpolated volume calculations.^17^ To date, there is no study accurately mapping the untreated, preoperative tumor growth of glioblastomas in vivo by using MRI with a maximum slice thickness of 1 mm. Furthermore, in the studies cited above, an exponential tumor growth was assumed for the calculation of the growth rate. Apart from mathematical models, studies demonstrating such exponential growth in untreated glioblastoma in humans do not exist either. Therefore, our first aim was to visualize preoperative tumor growth behavior and associated growth curves using tumor volumes of patients with at least 3 preoperative MRI scans. Depending on these results, we aimed to determine an underlying growth function (exponential, linear, etc.) in order to form the basis for further calculations. Subsequently, our second aim was to define the individual growth rate of tumors and to investigate whether there are influencing clinical parameters. Finally, our third aim was to analyze a possible association of the preoperative growth rate with OS of patients using multivariable analysis that included established predictors of postoperative survival in glioblastoma.

## Patients and Methods

### Patients

This retrospective study comprised all adult patients who underwent primary surgical resection or biopsy of a glioblastoma, IDH-wild type, WHO-grade 4 (diagnosis according to the CNS WHO 2021 classification^18^) between 2010 and 2020 at the Department of Neurosurgery, University Hospital Tübingen. Medical records of 749 patients were screened. We have searched the Picture Archiving and Communication System for the availability of patients with two preoperative MRI datasets with a maximum slice thickness of 1 mm, T1-weighted contrast-enhanced (CE) images and an interval of 7 days or longer between scans to be included for volumetric analysis (further called MRI1, MRI2, etc. chronologically). For further analysis, a subgroup of patients with the availability of 3 or more preoperative MRI datasets with CE tumor was created. The study protocol was approved by the local ethics committee (875/2021BO2).

### Patient Data and MRI Data

Clinical and demographic parameters, as well as tumor-specific data, were collected from patients’ medical records. IDH status was determined by immunohistochemistry, MGMT-promoter methylation status was determined by methylation-specific polymerase chain reaction or pyrosequencing assays for methylation of CpGs 74–78. Eloquence of localization was defined using the criteria by Chang et al.^19^ All patients included had postoperative CE MRI within 72 hours for the evaluation of EOR. The definition of residual tumor classification was based on the cutoff values (contrast enhancement) proposed by the RANO resect group^20^: Biopsy: No tumor reduction; Subtotal resection (STR): < 95% resection; near-total resection (NTR): 95%–99% resection; gross-total resection [GTR]): 100% resection of enhancing tumor.

All volumetric analyses of tumors were conducted by the first author (DF) and controlled by the senior author (CR) using the semi-automatic segmentation tool of 3DSlicer (http://www.slicer.org) as previously described.^21^ Volumetric analysis was performed for T1-weighted contrast-enhancing (T1-CE) tumors, total tumor volume was defined as T1-weighted CE tumor including, if present, cystic or necrotic portions. Three volumes were measured separately: Total tumor volume, contrast-enhancing part, and necrotic part.

### Statistical Analysis

Two consecutive steps were performed, with the second one being based on the results of the first:

*Step 1—Growth type*: Identification of an underlying growth type in patients with ≥3 preoperative MRI scans.

For visualization of tumor growth, absolute tumor volumes were plotted on a coordination system and regression curves of exponential and linear growth and the respective coefficient of determination *R*^2^ were created. Subsequently, curve estimation regression of all relative tumor volumes was conducted and possible growth functions (exponential, linear, cubic, and quadratic) were compared.

*Step 2—Growth rate*: Analysis of the preoperative growth rate of all patients with at least two scans fulfilling the abovementioned quality criteria.

Given both the results obtained in the first step and previously published studies,^14,22^ tumor growth was considered to be exponential in between MRI scans. Growth rate was calculated as previously described as VDT (in days) and specific growth rate (SGR; in percent per day).^14,23,24^

SGR and VDT were calculated using the following formulas:


$$\begin{array}{l} SGR=\frac{Ln\left(\frac{V(MRI2)}{V(MRI1)}\right)}{\Delta t}\;\;\; \end{array}$$

$$\begin{array}{l} VDT=\frac{Ln(2)}{SGR} \end{array}$$

V, volume in mm³; MRI1/2, 1^st^/2^nd^ MRI scan; Δt, time interval between scans in days. Mean SGR was used to calculate equivalent VDT (eVDT), which was supposed to give a more accurate value for average growth rate than mean/median VDT, which may overestimate results.^25^

All statistical analyses were conducted using IBM® SPSS® Statistics version 28.0 (IBM Corp., Armonk, NY, USA). For statistical tests, a *P*-value < .05 was considered statistically significant.

Tumor volumes and calculated growth rates are analyzed descriptively. A possible correlation of SGR with clinical parameters was calculated using the Pearson correlation coefficient (r), the Spearman correlation coefficient (rho), and point biserial correlation for parametric, non-parametric or dichotomous samples, respectively.

For further evaluation, we dichotomized SGR by using the median to groups of “fast ” and “slow”-growing tumor types. Categorical data were given in terms of absolute and percentage frequencies. For metric variables, either median and ranges or mean ± standard deviation were reported. Group differences were compared using Pearson’s χ2-test for categorical data and *t*-test, U-test, or Kruskal–Wallis test for continuous data. The representation of group-specific long-term survival was performed by means of Kaplan–Meier estimators. Group differences were calculated by applying the log-rank test.

Cox-proportional hazard regression model were carried out to assess the relationship between the factor “SGR” and survival stratified for different subgroups within the study cohort. Other included variables for further multivariable analysis (MGMT-promoter methylation status; extent of resection [EOR]; patients’ age) were selected based on their established prognostic value regarding survival in glioblastoma.^26,27^

## Results

### Step 1—Observation of an Exponential Growth Type

To identify possible growth patterns of glioblastoma, patients with ≥3 preoperative cerebral MRI scans were analyzed. Thirteen patients were included, 11 with 3, and 2 patients with 5 imaging timepoints, each. All but one of these patients had a tumor volume < 3 cm³ (median: 0.68 cm³; range: 0.10 cm³–39.96 cm³) on the first MRI. The longest time interval between first and last imaging was 133 days, the shortest was 23 days.

Scatter plots of absolute values of tumor volume and days between imaging were generated to visualize tumor growth. Curve plots of volume change of 12 of the 13 included tumors visually showed an exponential growth pattern with better fit of the corresponding regression curve (Supplementary Figure 2). A curve estimation regression including all relative tumor volumes showed the best fit for the exponential curve among the growth functions tested (linear, exponential, cubic, and quadratic; Supplementary Figure 3).

**Figure 1.**
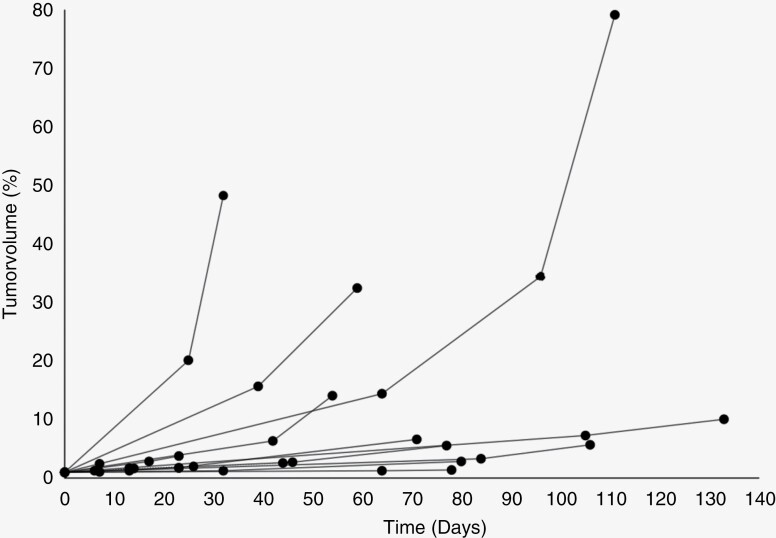
Evolution over time of relative tumor volumes in 13 patients with untreated glioblastoma with ≥3 preoperative MRI.

**Figure 2.**
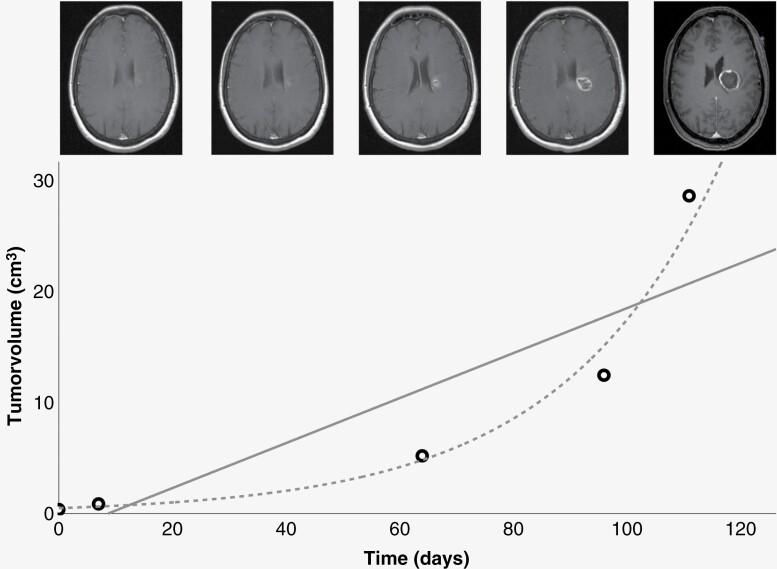
Example of exponential growth pattern. Tumor volume per time of a patient, initially diagnosed with multiple sclerosis and biopsied ~110 days after first MRI. Tumor volumes are depicted as circles, curve estimation regression for exponential and linear growth is shown in gray. Corresponding contrast-enhanced-enhanced cerebral MRI imaging is depicted for each tumor volume. Exponential curve estimation shows significant fit (*R*² = 0.975, *P* = .001).

Relative volumes of all tumors and imaging timepoints included in this analysis can be found in Figure 1. Although most tumors showed an exponential growth pattern when examined individually, growth rates differed greatly among different patients. The coefficient of determination *R*² for the respective exponential functions was between 0.93 and 0.99. Tumor growth rate showed strong variability, with VDT between 5.7 and 192.5 days. Reasons for multiple preoperative MRI examinations varied among patients, such as initial wrong differential diagnosis or postponement of surgery at patients’ request. An exemplary case of a patient with a total of 5 MRI examinations and exponential growth can be found in Figure 2.

### Step 2—Descriptive Statistics of Preoperative Growth Rate, Analysis of Correlating Factors, and Association With Survival

In a second step, we analyzed the individual preoperative VDT and SGR of patients with 2 preoperative MRI scans with 1 mm slice thickness, at least 7 days apart. 70 patients met the inclusion criteria and were included for further analysis.

Table 1 shows different tumor volumes of the preoperative MRI scans and corresponding calculated growth rates and parameters.

**Table 1.** Descriptive *Statistics* of Preoperative Tumor Growth

	Total tumor (T1 contrast-enhanced)	CE	Necrosis
Volumes			
1^st^ preoperative MRI			
Mean (cm³)	17.5 ± 21.0	7.8 ± 8.1	5.4 ± 9.4
Median (cm³; min; max)	9.9 (0.1; 83.2)	5.3 (0.1; 38.4)	1.2 (0; 40.2)
Fraction of total tumor volume (%)	1	60	19
2^nd^ preoperative MRI			
Mean (cm³)	22.7 ± 24.6	10.2 ± 10.3	7.4 ± 11.5
Median (cm³; min; max)	13.7 (0.8; 98.9)	6.7 (0.8; 55.6)	2.4 (0; 46.8)
Fraction of total tumor volume (%)	1	56	22
Absolute growth (cm³)			
Mean (cm³)	5.2	2.4	1.9
Median (cm³)	3.7	1.7	0.7
Relative growth (%)	34.2	46.3	55.9
*Growth parameters*			
VDT (days; median; min; max)	31 (−42; 3013)	16 (−623; 3414)	18 (−697; 918)
SGR (%/ day)			
Median; min; max	2.2 (−2.6; 18.4)	2.4 (−12.2; 17.7)	3.1 (−2.6; 59.6)
Mean	2.9 ± 3.4	2.8 ± 4.6	5.2 ± 8.5
eVDT (days)	24	24	13
Days between MR images (days; median; min; max)	14 (7; 110)		

Median tumor volume was 9.9 cm³ at the time of the first scan (range 0.1–83.2) and 13.7 cm³ (0.8–98.9) at the second one. Median relative tumor growth was 34.2% between imaging timepoints. The median VDT was 31 days (−42–3013). The median SGR was 2.2 % (range −2.6–18.4) and mean SGR was 2.9 % (± 3.4). eVDT calculated from mean SGR was 24 days. Two tumors showed a volumetric “shrinkage” in between scans. Over time, the necrotic part generally showed an increase in proportion, while CE showed a decrease: The mean percentage of necrosis of the tumor was 19% on the first preoperative scan and increased to 22% on the second scan. This can also be seen in the relative growth estimation with 46.3% for the CE versus 55.9% for the necrotic part.

SGR correlated negatively with tumor size seen on the first MRI (Spearman: rho = −0.59, *P* < .001). There was a positive correlation in patients with a high ratio of CE to total tumor volume (Spearman: rho = 0.30, *P* = .01), as well as a negative correlation of SGR in patients with a large necrotic tumor portion (Spearman: rho = −0.44, *P* < .001). No correlation was found for age, sex, MGMT-promoter status, or contact with the dura (Supplementary Figure 4/ Table 1).

### Definition of Growth Types and Analysis of Associated Characteristics

All patients were dichotomized into “fast” and “slow” growth types based on the median SGR. Clinical and surgical features of all patients and the growth types can be seen in Table 2.

**Table 2.** Characteristics of Growth Groups “Slow Type” and “Fast Type”

Parameter	All patients included	Patients defined by tumor growth type		*P*-value
	(*n* = 70)	Fast type (*n* = 35)	Slow type (*n* = 35)	
Age (years)	63 ± 11	66 ± 12	61 ± 11	.10 ^**+**^
*Sex*				
male	40/70 (57%)	22/35 (63%)	18/35 (51%)	.33 ^**x**^
*Type of surgery*				**.04** ^**x**^
Resection	46/70 (66%)	19/35 (54%)	27/35 (77%)	
Biopsy	24/70 (34%)	16/35 (46%)	8/35 (22%)	
OS (months)	14.8 ± 13.1	11.3 ± 8.8	18.3 ± 15.9	**.02** ^**+**^
PFS (months)	7.8 ± 8.4	6.1 ± 6.5	9.5 ± 9.8	.10 ^**+**^
*Tumor location*				
Left side	42/70 (60%)	21/35 (60%)	21/35 (60%)	1 ^x^
Eloquent	33/70 (47%)	19/35 (54%)	14/35 (40%)	.23 ^x^
Contact with dura	27/70 (39%)	11/35 (31%)	16/35 (46%)	.22 ^x^
MGMT-Promoter methylation	29/70 (41%)	15/35 (43%)	14/35 (40%)	.81 ^x^
Preoperative KPS (median; quartiles)	90 (70–90)	90 (70–90)	90 (80–90)	.66 ^x^
Preoperative corticosteroids	22/70 (31%)	5/35 (14%)	17/35 (49%)	**<.01** ^x^
First line treatment Second line treatment				.46 ^x^
Radiotherapy (RT)	17/70 (24%)	10/35 (29%)	7/35 (20%)	
Hypofractionated RT	10/17 (59%)	5/10 (50%)	5/7 (71%)	
Temozolomid + RT		1/10	0/7	
Temozolomid + RT		0/10	1/7	
Re-resection and Temozolomid		1/10	0/7	
Re-resection and Stupp regimen		0/10	1/7	
Temozolomide	4/70 (6%)	2/35 (6%)	2/35 (6%)	
Involved fields RT		1/2	0/2	
Bevacizumab		1/2	0/2	
Re-resection and Stupp regimen		0/2	1/2	
Radiochemotherapy with temozolomide (Stupp regimen)	36/70 (51%)	19/35 (54%)	17/35 (49%)	
Involved fields RT		1/19	0/17	
Temozolomid		1/19	3/17	
Lomustin		6/19	5/17	
Involved fields RT + Lomustin		1/19	0	
Re-resection		0/19	1/17	
Re-resection and Stupp regimen		1/19	0/17	
Re-resection and Lomustin		1/19	1/17	
No tumor-specific therapy/best supportive care	4/70 (6%)	1/35 (3%)	3/35 (9%)	
Unknown	4/70 (6%)	2/35 (6%)	2/35 (6%)	
MS CA209–548 study	1/70 (1%)	0/35	1/35 (2%)	
CeTeG study	3/70 (4%)	0/35	3/35 (9%)	
Re-resection		0	1/3	
Re-resection and Temozolomid		0	1/3	
GLARIUS study	1/70 (1%)	1/35 (3%)	0/35	
Re-resection		1/1	0	
Absolute tumor volume MRI1 (c)		9.5 ± 16.0	25.5 ± 22.9	**<.001** ^**+**^
Absolute tumor volume MRI2 (cm³)		15.8 ± 21.2	29.5 ± 26.0	**<.001** ^**+**^
Proportion of CE in MRI1 (%)		68 ± 21	50 ± 23	**.02** ^**+**^
Proportion of necrosis in MRI 1 (%)		13 ± 13	25 ± 16	**.01** ^**+**^
VDT (median)		60	19	**.02** ^u^
SGR (mean)		0.8	5.0	**<.001** ^**+**^
Subgroup tumor resection (*n* = 46)				
		Fast type (*n* = 19)	Slow type (*n* = 27)	*P*-value
Age (years)		61 ± 11	61 ± 12	.94 ^+^
OS (months)		14.4 ± 9.8	21.6 ± 15.8	.07 ^u^
PFS (months)		6.9 ± 8.1	10.9 ± 10.6	**.04** ^u^
Extent of resection				.78 ^x^
GTR		7/19 (37%)	12/27 (44%)	
NTR		9/19 (47%)	10/27 (37%)	
STR		3/19 (16%)	5/27 (19%)	
Reoperation during follow-up		4/19 (21%)	7/27 (26%)	.70 ^x^
Time until reoperation (days)		231 ± 165	367 ± 320	.23 ^u^
MGMT-promoter methylation		9/19 (47%)	10/27 (37%)	.48 ^x^
First line treatment Second line treatment				.44 ^x^
Radiotherapy		5/19 (26%)	5/27 (19%)	
Temozolomid + RT		0/5	1/5	
Temozolomid		1/5	0/5	
Re-resection and Temozolomid		1/5	0/5	
Re-resection and Stupp regimen		0/5	1/5	
Temozolomide		2/19 (10%)	2/27 (7%)	
Involved fields RT		1/2	0/2	
Bevacizumab		1/2	0/2	
Re-resection and Stupp regimen		0	1/2	
Radiochemotherapy with temozolomide (Stupp regimen)		10/19 (53%)	15/27 (56%)	
Involved fields RT		1/10	0/15	
Re-resection		0/10	1/15	
Lomustin		2/10	4/15	
Temozolomid		1/10	2/15	
Re-resection and Stupp regimen		1/10	0/15	
Re-resection and lomustin		1/10	1/15	
No tumor-specific therapy/best supportive care		0/19	2/27 (7%)	
Unknown		1/19 (5%)	0/27	
MS CA209-548 Study		0/19	1/27 (4%)	
Re-resection		0	1/1	
CeTeG study		0/19	2/27 (7%)	
Re-resection		0	1/2	
GLARIUS study		1/19 (5%)	0/27	
Re-resection		1/1	0	

^x^, Pearson’s χ2-test; ^+^, *t*-test; ^u^, U-test; ClinicalTrials.gov ID of studies = MS CA209-548: NCT03233152, CeTeG: NCT01149109, and GLARIUS: NCT00967330.

The mean age at surgery was 63 ± 11 years. Forty-six patients (66 %) were treated with tumor resection, 24 had a biopsy without further tumor resection. Of all patients with tumor resection, 11 patients underwent reoperation for tumor recurrence during follow-up (median time till reoperation = 13 months). Mean follow-up was 14.8 ± 13.1 months.

The analysis of the entire cohort revealed that OS was significantly longer in the slow, compared to the fast-growth type group (18.3 vs. 11.3 months, *P* = .02). Progression-free survival (PFS) was longer in this group as well, but the results did not reach statistical significance (9.5 vs. 6.1 months, *P* = .10). Tumor volumes on initial MRI differed significantly between both groups with 9.5 cm^3^ in the fast versus 25.5 cm^3^ in the slow-growth type (*P* < .001) and 15.8 cm^3^ vs. 29.5 cm^3^ (*P* < .001) on the second preoperative MRI, respectively. Patients with the slow-growth type (17 out of 35 patients) and larger mean tumor volumes were more likely to receive corticosteroids preoperatively than patients of the fast-growth type (5 out of 35 patients; *P* = .002). MGMT-promoter methylation status was available in all patients, 29 had methylated promoters, which were distributed equally between both cohorts (*P* = .81). There were also no significant differences for tumor localization, eloquence, and postoperative therapy. Faster-growing tumors were more likely to receive biopsy than resection (*P* = .04). Median VDT and mean SGR were 60 days and 0.8% for the slow-growth and 19 days and 5.0% for the fast-growth group, respectively, (*P* = .02 and *P* < .001).

### Subgroup Analysis After Tumor Resection

Additionally, we have performed subgroup analysis of patients after tumor resection (*n* = 46). OS was also longer in the slow-growth type group, but the result did not reach statistical significance (21.6 vs. 14.4 months, *P* = .07). PFS was significantly longer in this group (10.9 vs. 6.9 months, *P* = .04). No statistical difference was found between the growth groups for EOR (*P* = .78), reoperation during follow-up (*P* = .70), time until reoperation (*P* = .23), MGMT-promoter methylation status (*P* = .48) or postoperative therapy (*P* = .44).

### Association of Growth Patterns With Postoperative Survival

**Figure 3.**
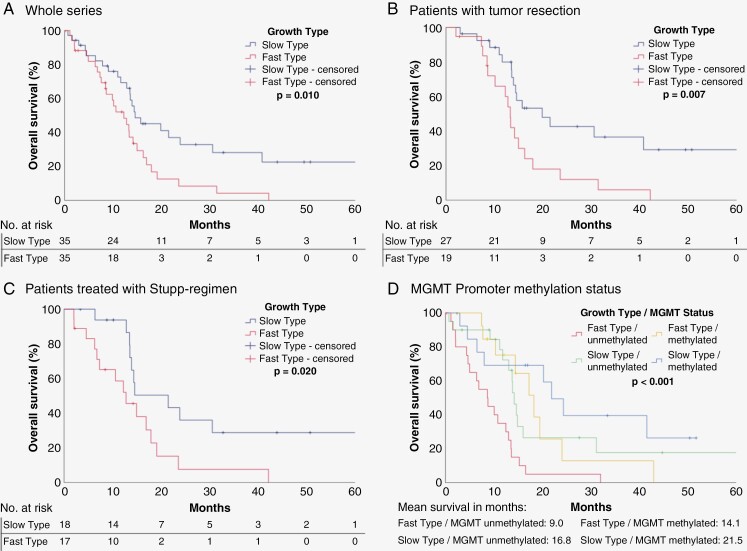
Kaplan–Meier Curves for the growth types “Slow Type” and “Fast Type” for different subgroups. (A) All patients (B) Patients after tumor resection (C) Patients treated with radiochemotherapy with temozolomide (D) MGMT-promoter methylation status

Figure 3 The slow type group showed a significantly longer OS compared to the fast one (figure 3A; log-rank: *P* = .010). This effect seemed to be time-dependent as it was mainly seen in long-term survival. Subanalysis showed that especially after microsurgical tumor resection, patients in the slow-growth group had an OS advantage over the fast-growth group. (Figure 3B; log rank: *P* = .007). Comparable OS advantages were seen for the subgroup of patients after radiochemotherapy with temozolomide^5^ (Figure 3C). A subanalysis (Figure 3D) showed that the presence of both MGMT-promoter methylation and a slow-growth type was beneficial for overall survival, whereas fast-growth and absence of methylation were associated with the shortest life expectancy, the other 2 in-between groups did not show a major difference in long-term survival (mean survival in months: 9.0 vs. 14.1 vs. 16.8 vs 21.5, log rank: *P* < .001, Kruskal–Wallis test: *P* = .03). In the subgroup of patients after biopsy only, there was no statistically significant difference between both growth types, yet these groups were rather small with 16 and 8 patients each. The analysis of PFS also revealed advantages for the slow-growth type as seen on the Kaplan–Meier curves, although the level of statistical significance was not achieved (Supplementary Figures 5 and 6).

Univariable Cox-regression models stratified for the respective groups (all patients, patients with tumor resection, patients with tumor biopsy) showed a significant influence of SGR on survival for patients with tumor resection, while no significant interaction was observed for the other (Supplementary Tables 2–4). An interaction between preoperative tumor size and SGR was examined in a regression including both variables; SGR remained a significant predictor of survival, while no significant interaction with OS was found for preoperative tumor size (Supplementary Table 5).

### Multivariable Analysis

The multivariable Cox-regression model stratified for patients with resection of glioblastoma (table 3) demonstrated that SGR was independently associated with overall survival (HR: 1.25, 95% CI: 1.10; 1.43, *P* < .001). The variable “EOR” was also a significant predictor of OS. In this regard, GTR appeared to have a significant advantage over NTR and STR for this cohort as well. Patients’ age was at the level of statistical significance (HR: 1.04; 95% CI: 1.00; 1.07, *P* = .05), while MGMT-promoter methylation status did not reach statistical significance in this small subgroup (HR: 0.75, 95% CI: 0.35; 1.63, *P* = .47). As indicated in univariable models, SGR alone was not significant as a predictor for survival in a multivariable model for the whole series performed additionally (Supplementary Table 6)

**Table 3.** Multivariable Cox Proportional-Hazards Regression for the Subgroup of Patients With Tumor Resection

Characteristic	HR	95% CI	*P*-value
SGR ^a^	1.25	1.10; 1.43	**<.001**
MGMT-promoter methylation status	0.75	0.35; 1.63	.47
Age	1.04	1.00; 1.07	.05
Extent of resection			**.04**
STR	Reference		
NTR	0.39	0.17; 1.18	.09
GTR	0.23	0.07; 0.71	**.01**

HR, hazard ratio, CI, confidence interval, ^a^ multiplied by 100. Significant P-values are written in bold letters.

## Discussion

The first step of this retrospective study was to identify a growth type in untreated patients with glioblastoma who received ≥3 preoperative MRI scans and therefore contribute to the understanding of the natural growth of glioblastomas.

Most patients visually exhibited exponential tumor growth, which could be confirmed in a regression analysis. This is especially true for rather small tumors. In particular, this exponential growth type could be visualized well in patients with 5 preoperative MRI timepoints, initially misdiagnosed as multiple sclerosis (exemplary case, Figure 2). Interestingly, our results are consistent with a recently published study showing exponential glioblastoma growth in a preclinical glioma mouse model.^13^ Despite the exponential growth pattern of the tumors, strongly varying growth rates were found. Our analysis showed that growth rates significantly correlated with tumor size. Possible reasons for this have been discussed in the past, but several assumptions must be made: One is that tumors might decelerate growth as a natural limitation caused by larger size, large necrosis, and areas of insufficient supply of nutrition.^14^ On the other hand, it remains unclear why some tumors are detected late when they are already large, and others are detected very early. As fast-growing tumors tended to be detected early with only small size in this study, it might be speculated that this is caused by fast-growing tumors becoming symptomatic at an earlier point. In this regard, Young et al. recently described a significant association between greater SGR and lower preoperative KPS.^16^ In this case, the brain may not have enough time to adjust to the local growth-related changes and possibly local pressure may occur as a result. This might be seen in contrast to slow-growing tumors, which become symptomatic at a rather late stage with already impressive extension of size.

Due to the correlation of tumor size with growth rate, we concluded that glioblastoma growth may indeed exhibit Gompertzian growth (or logistic) when considered as a whole, as previously assumed.^14^ However, patients are usually treated soon after the initial diagnosis, therefore tumors may still be in a phase of logistic or Gompertzian growth, in which near-exponential growth is present. In such cases, the tumor growth likely has not reached a plateau yet and represents an early stage of tumor growth.^28^ Beyond that, although a plateau in tumor growth can be expected, it remains unclear when and at what tumor size this will eventually occur. Consequently, calculation of VDT and SGR may be appropriate for assessing growth rates in glioblastoma, especially in the immediate preoperative period and for “smaller” tumors.

The second step of this study was to analyze factors influencing the preoperative growth rate and its prognostic impact on OS and PFS. To the best of our knowledge, this study is the first to investigate such precise volumetrics by two preoperative MRIs with 1 mm MRI-slice-thickness exclusively. Based on our results, we assumed exponential growth, and therefore used VDT and SGR as descriptions of growth. We found a median VDT of 31 days, a median SGR of 2.2% and a mean SGR of 2.9%, with a respective eVDT of 24 days. Previously reported values of VDT and SGR range from 21.1 days to 46.8 days and 1.1 % to 2.1%, respectively.^8,14,22,29^ Most cited studies only reported median values, as the growth rate had great ranges. Nevertheless, results of our study coincide with the reported values and show that high-grade gliomas can grow rapidly with doubling times of less than one month. This underlines the importance of maintaining a close patient follow-up as well as rapid referral for further diagnostics and therapy.

In our analysis with high-quality MRI data, we could confirm the previous finding that especially tumors presenting small in the first MRI tend to show a higher growth rate than large ones.^14^ These results might have direct consequences for neurosurgical operation planning in the future, as special emphasis should be placed on updated MR imaging preoperatively in these cases. Furthermore, the initiation of therapy should be considered time-critical. Although a recent multicentric study indicated that there is no significant interaction between outcome and time-to-surgery,^8^ we think that clinicians should not be wrongly reassured by a small tumor size, possibly postponing an operation based on the argument that the tumor size is still relatively small. Apart from tumor size and portion of CE, preoperative steroid administration showed negative correlation with growth rate, 2 tumors showed reduction in their volume between scans. This effect has previously been shown and possibly reflects a dependency between tumor size and steroid administration likely based on reduced swelling.^14^ We were not able to identify other factors explaining the differences in growth rates. In this regard, further molecular evaluations may be necessary to possibly identify new relevant markers that might be targeted in the future.

Although glioblastoma is arguably a whole-brain disease,^30^ analysis of the growth dynamics of the “measurable” part of the tumor in CE T1 sequences showed a favorable impact of slow preoperative growth on postoperative long-term survival in Kaplan–Meier Curves. This benefit was seen mainly in long-term survivors, which has been described before.^15^ In further multivariable analysis, an overall survival benefit for slower preoperative growth rate could only be shown for patients with surgical tumor mass reduction. No significant result for SGR was found for the whole series, as not all of these patients were treated with the highly important prognosticator “surgical resection.” In particular, the subgroup of patients receiving tumor biopsy and adjuvant radio-/chemotherapy did not show any evidence of interaction between survival and preoperative growth rate at all (no tumor that was biopsied underwent secondary resection in this particular study sample). This lack of significance of the growth rate in patients who were only biopsied possibly affected the statistical analysis for the whole series and might be due to an already pre-existing reduced general condition and also the life expectancy of these patients, thus the growth rate had no relevance on survival. On average, patients in this group were considerably older, and thus potentially were not capable of standard treatment options. As shown in Kaplan–Meier Curves (Figure 3B), a survival benefit for the group with slower preoperative growth could not be observed immediately postoperatively, but after a considerable period of time. For the subgroup of resected patients, this effect was independent of other established predictors of postoperative survival, such as age, MGMT status, or EOR. In this multivariable analysis, GTR was a positive predictor for OS, while any residual tumor (NTR) did not reach statistical significance over a subtotal resection. This coincides with the results of a recently published, large multicentric study by Roder et al and older data by Stummer et al.^31,32^

Furthermore, the combination of both slower preoperative growth and MGMT-promoter methylation was shown to be associated with the most favorable overall survival. Interestingly, patients with fast preoperative growth type and methylated MGMT-promoter showed comparable survival to patients with slow preoperative growth and unmethylated promoter (Figure 3D). This underlines the potential value of preoperative growth rates as a prognostic parameter for long-term survival in glioblastoma, similar observations have been made in other tumor entities.^33–35^

This study has several limitations: A main limitation is the retrospective and single-center design of this study. A prospective data acquisition of repetitive MRI in untreated human glioblastoma over a longer period of time to visualize the natural growth might not be possible for ethical reasons, hence this study had to rely on data of patients who were scheduled for multiple serial MRI before surgery without treatment in between. These patients may correspond to a subgroup of patients which is not representative for the whole glioblastoma population. Furthermore, the resulting rather small size of the study cohort limited statistical analysis, especially for multivariable models. For a multivariable analysis accounting for other variables such as (postoperative) first-line therapy, further studies with a larger number of patients are required.

Particularly tumors of patients included in step 1 mostly showed a slow-growth rate and were rather small in the first MRI. It is therefore questionable to which extent these results can be transferred from this group to the cohort as a whole. This might also apply to the correlation of growth rate with tumor size. In order to avoid a possible bias due to variability in initial tumor size, future studies should therefore compare growth rate of similar-sized and located tumors at first time of diagnosis.

Although we only included high-quality images with a maximum slice thickness of 1 mm, other parameters like field strength, different types of scanners, or timing of contrast application might also influence image evaluation.^36^ Furthermore, the tumor measurements are limited to the radiologically visible, contrast-enhancing, and necrotic parts. The infiltrative and non-CE parts of the tumor cannot be measured in the method that was used and is therefore not considered.

## Conclusion

This study demonstrated near-exponential tumor growth in untreated human glioblastoma in vivo in the preoperative period. Furthermore, analysis of growth rates based exclusively on MRIs with 1-mm slice thickness showed highly variable growth rates with a tumor doubling time of 31 days. The preoperative growth rate was significantly associated with overall survival after surgical resection, independent of additional factors. With these findings, additional subclassifications and molecular analysis with predictive value and possible new treatment targets might be possible in the future after verification in large prospective multicenter cohorts.

## Supplementary Material

vdae053_suppl_Supplementary_Material

## Data Availability

De-identified data generated or analyzed during this study are available from the corresponding author upon reasonable request.
