# Supplementary material for: Preoperative growth dynamics of untreated glioblastoma: Description of an exponential growth type, correlating factors, and association with postoperative survival
Source: Neurooncol Adv. 2024 Apr 3;6(1):vdae053. doi: 10.1093/noajnl/vdae053 (PMC11046984; doi:10.1093/noajnl/vdae053)
Supplement: vdae053_suppl_Supplementary_Material [file vdae053_suppl_supplementary_material.docx]

Supplementary Figure 1: Flow-chart of patient selection


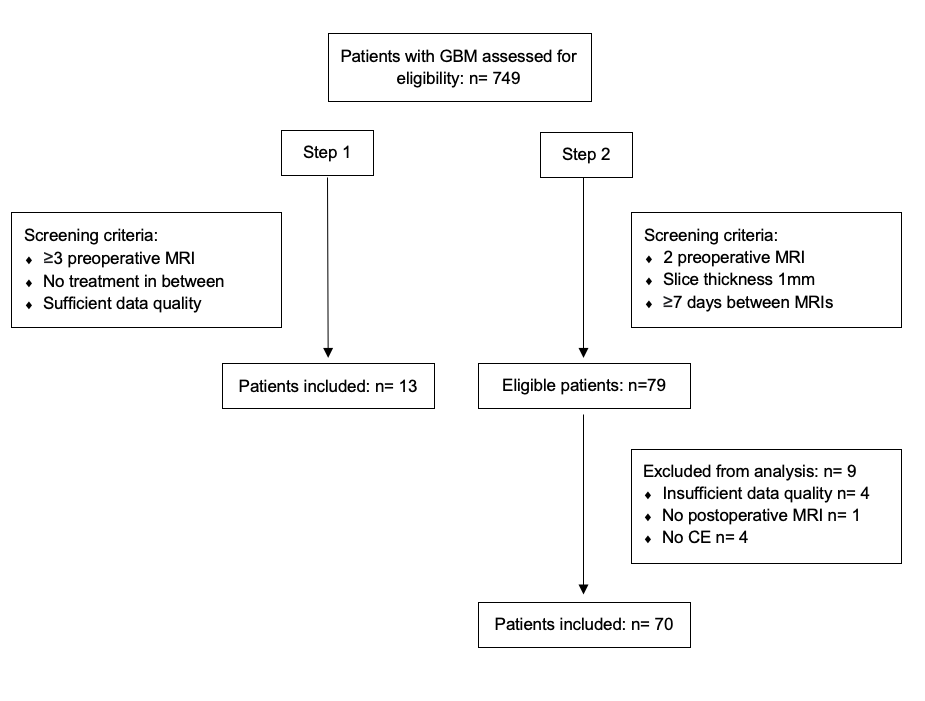


Supplementary Figure 2: Scatter plots of tumor volume (black circles) of 13 included patients. Curve estimation regression displayed in grey for linear and exponential growth with corresponding (corrected) R^2^ – value and p-value.

y-axis: tumor volume in cm³; x-axis: time in days

Supplementary Figure 3: Curve estimation regression for relative tumor volumina (black circles) of all 13 patients for different possible growth functions: A = exponential; B = linear; C = cubic; D = quadratic

y-axis: tumor volume in %; x-axis: time in days

Supplementary Figure 4 / Table 1: Correlation of SGR with clinical parameters


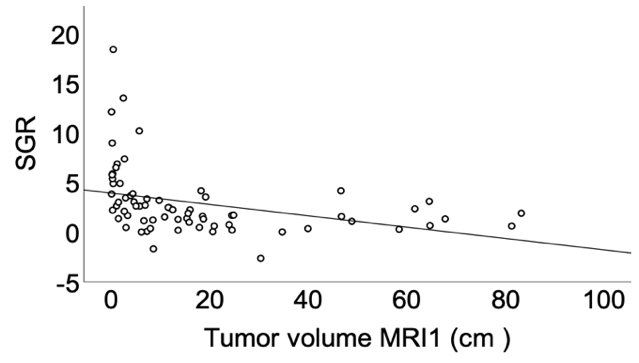

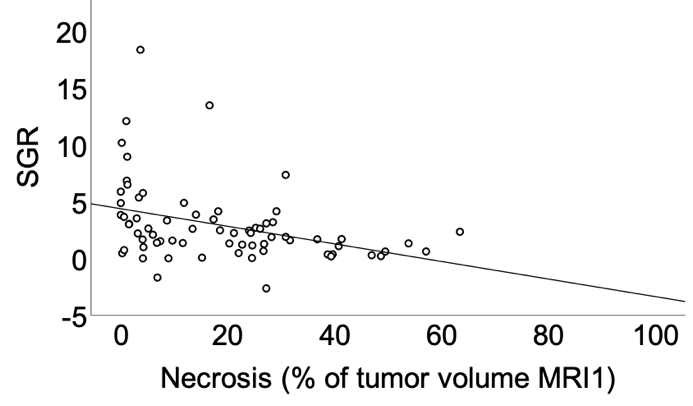

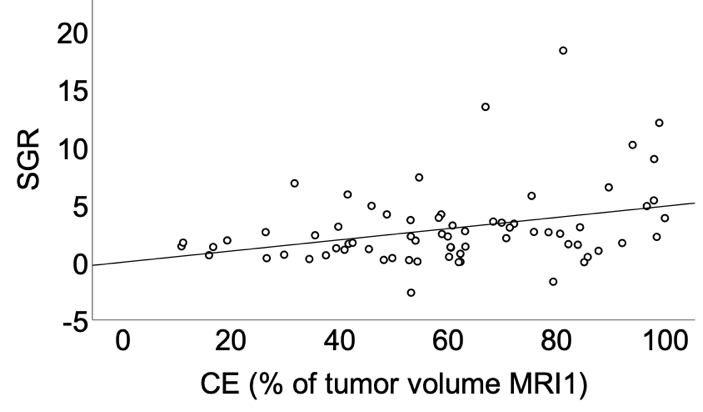

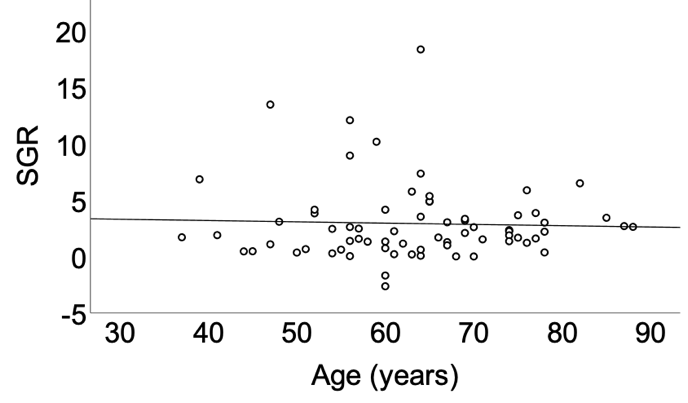


| Parameter | Correlation coefficient rho / r | *p*-value |
| --- | --- | --- |
| Tumor volume MRI1 | -0.59 | <0.001 |
| Necrosis (% of tumor volume MRI1) | 0.30 | 0.01 |
| Age |  |  |
| CE (% of tumor volume MRI1) | -0.44 | <0.001 |
| Sex | 0.07 | 0.55 |
| MGMT Promoter Methylation | 0.06 | 0.61 |
| Preoperative corticosteroid administration | -0.40 | <0.001 |
| Dura contact | -0.17 | 0.16 |

Supplementary Figure 5: Kaplan-Meier Curve for survival for Patients with tumor biopsy and adjuvant treatment


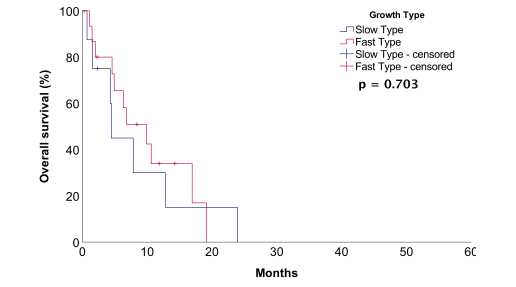


Supplementary Figure 6: Kaplan-Meier Curve for postoperative Progression-free survival for patients with tumor resection
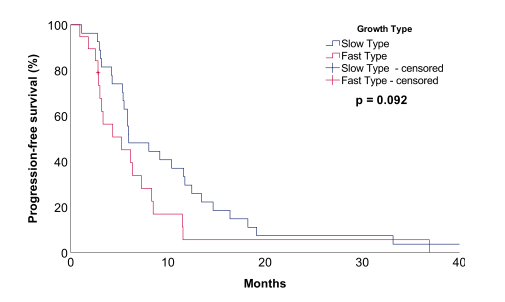


Supplementary Figure 7: Kaplan-Meier Curve for Extent of Resection, subgroups GTR, NTR, STR and biopsy


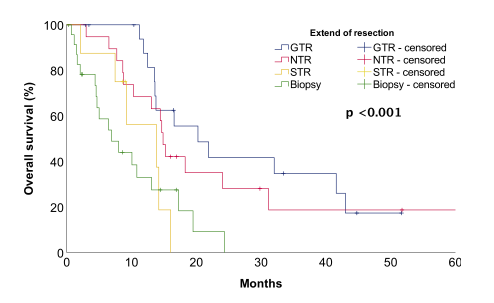


Supplementary Figure 8: Kaplan-Meier Curve for MGMT-promoter methylation status – All patients


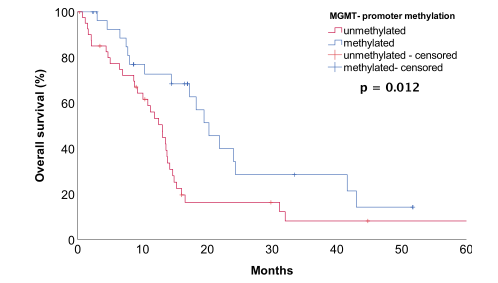


Supplementary Figure 9:

Kaplan-Meier Curve for MGMT-promoter methylation status – Patients with tumor resection


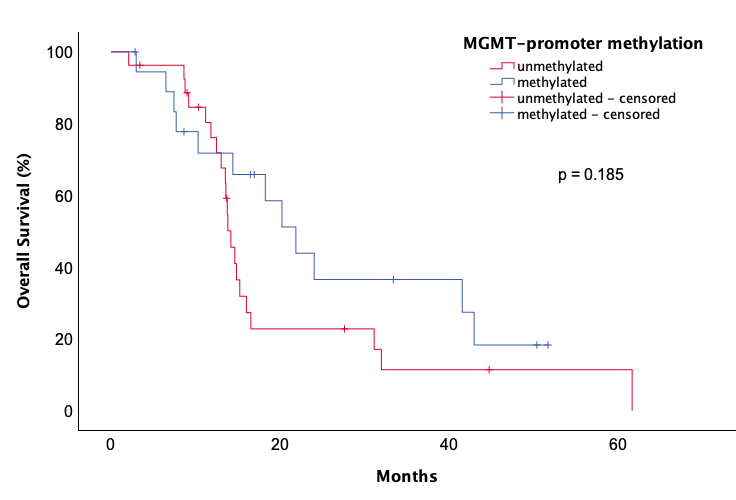


Supplementary Tables 2-4: Univariable Cox regression models for the variable “SGR” stratified for different subgroups within the study cohort

All patients

|  | HR | 95,0% CI | | p-value |
| --- | --- | --- | --- | --- |
|  |  | Lower | Upper |  |
| SGR | 1.07 | 0.99 | 1.15 | 0.100 |
|  |  |  |  |  |

Subgroup patients with tumor resection

|  | HR | 95,0% CI | | p-value |
| --- | --- | --- | --- | --- |
|  |  | Lower | Upper |  |
| SGR | 1.21 | 1.07 | 1.37 | 0.002 |
|  |  |  |  |  |

Subgroup patients with tumor biopsy

|  | HR | 95,0% CI | | p-value |
| --- | --- | --- | --- | --- |
|  |  | Lower | Upper |  |
| SGR | 0.90 | 0.78 | 1.04 | 0.15 |
|  |  |  |  |  |

Supplementary Table 5: Multivariable Cox regression (resected patients) for the variables SGR and preoperative Tumorvolume in patients with tumor resection; ^a^ = log-transformed

|  | HR | 95,0% CI | | p-value |
| --- | --- | --- | --- | --- |
|  |  | Lower | Upper |  |
| SGR | 1.21 | 1.07 | 1.37 | 0.003 |
| Preoperative tumorvolume ^a^ | 1.06 | 0.46 | 2.45 | 0.9 |

Supplementary Table 6: Multivariable Cox regression for the whole series

|  | HR | 95,0% CI | | p-value |
| --- | --- | --- | --- | --- |
|  |  | Lower | Upper |  |
| SGR | 1.06 | 0.97 | 1.15 | 0.24 |
| Age | 1.04 | 1.01 | 1.07 | 0.06 |
| MGMT promoter methylation | 0.32 | 0.16 | 0.63 | 0.001 |
| Extent of resection |  |  |  | 0.002 |
| Biopsy | Reference |  |  |  |
| STR | 0.19 | 0.08 | 0.45 | <0.001 |
| NTR | 0.35 | 0.15 | 0.78 | 0.01 |
| GTR | 0.46 | 0.16 | 1.34 | 0.16 |
| Sex | 1.09 | 0.58 | 2.10 | 0.79 |
